# The effect of activated protein C on experimental acute necrotizing pancreatitis

**DOI:** 10.1186/cc3485

**Published:** 2005-03-04

**Authors:** Levent Yamenel, Mehmet Refik Mas, Bilgin Comert, Ahmet Turan Isik, Sezai Aydin, Nuket Mas, Salih Deveci, Mustafa Ozyurt, Ilker Tasci, Tahir Unal

**Affiliations:** 1 Assistant Professor, Medical Intensive Care Unit, Gülhane School of Medicine, Etlik, Ankara, Turkey; 2 Associate Professor, Department of Internal Medicine, Gülhane School of Medicine, Etlik, Ankara, Turkey; 3 Associate Professor, Medical Intensive Care Unit, Gülhane School of Medicine, Etlik, Ankara, Turkey; 4 Resident, Department of Internal Medicine, Gülhane School of Medicine, Etlik, Ankara, Turkey; 5 Resident, Department of Surgery, Numune Training Hospital, Sihhiye, Ankara, Turkey; 6 Resident, Department of Anatomy, Medical Faculty of Hacettepe University, Sihhiye, Ankara, Turkey; 7 Assistant Professor, Department of Pathology, Gülhane School of Medicine, Etlik, Ankara, Turkey; 8 Associate Professor, Department of Microbiology, Gülhane School of Medicine, Etlik, Ankara, Turkey; 9 Assistant Professor, Department of Internal Medicine, Gülhane School of Medicine, Etlik, Ankara, Turkey; 10 Professor, Department of Internal Medicine, Gülhane School of Medicine, Etlik, Ankara, Turkey

## Abstract

**Introduction:**

Acute pancreatitis is a local inflammatory process that leads to a systemic inflammatory response in the majority of cases. Bacterial contamination has been estimated to occur in 30–40% of patients with necrotizing pancreatitis. Development of pancreatic necrosis depends mainly on the degree of inflammation and on the microvascular circulation of the pancreatic tissue. Activated protein C (APC) is known to inhibit coagulation and inflammation, and to promote fibrinolysis in patients with severe sepsis. We investigated the effects of APC on histopathology, bacterial translocation, and systemic inflammation in experimental acute necrotizing pancreatitis.

**Materials and method:**

Forty-five male Sprague-Dawley rats were studied. Rats were randomly allocated to three groups. Acute pancreatitis was induced in group II (positive control; *n *= 15) and group III (treatment; *n *= 15) rats by retrograde injection of taurocholate into the common biliopancreatic duct. Group I rats (sham; *n *= 15) received an injection of normal saline into the common biliopancreatic duct to mimic a pressure effect. Group III rats were treated with intravenous APC 6 hours after induction of pancreatitis. Pancreatic tissue and blood samples were obtained from all animals for histopathological examination and assessment of amylase, tumor necrosis factor-α, and IL-6 levels in serum. Bacterial translocation to pancreas and mesenteric lymph nodes was measured.

**Results:**

Acute pancreatitis developed in all groups apart from group I (sham), as indicated by microscopic parenchymal necrosis, fat necrosis and abundant turbid peritoneal fluid.

Histopathological pancreatitis scores in the APC-treated group were lower than in positive controls (10.31 ± 0.47 versus 14.00 ± 0.52; *P *< 0.001). Bacterial translocation to mesenteric lymph nodes and to pancreas in the APC-treated group was significantly decreased compared with controls (*P *< 0.02 and *P *< 0.007, respectively). Serum amylase, tumor necrosis factor--α, and IL-6 levels were also significantly decreased in comparison with positive controls (*P *< 0.001, *P *< 0.04 and *P *< 0.001, respectively).

**Conclusion:**

APC improved the severity of pancreatic tissue histology, superinfection rates and serum markers of inflammation during the course of acute necrotizing pancreatitis.

## Introduction

Acute pancreatitis is a local inflammatory process that leads to a systemic inflammatory response in the majority of the cases [1-3]. Severe and life-threatening complications requiring intensive care occur in about 25% of patients with acute necrotizing pancreatitis (ANP) [4]. While the intra-acinar premature activation of digestive enzymes is central to pathophysiological mechanisms of injury, acinar cell apoptosis, increase in oxidative stress, microcirculatory derangements, and release of cytokines contribute to progression of injury and development of extrapancreatic complications [1-5]. Severe acute pancreatitis is usually a result of glandular necrosis [6]. Nuclear factor-κB (NF-κB), a transcription factor that is associated with immediate early gene activation, plays a critical role in the development of necrosis. Although the exact mechanism of NF-κB activation is unknown, once stimulated it leads to production of several inflammatory cytokines, including tumor necrosis factor (TNF)-α [7]. This cytokine is known to increase the severity of pancreatitis by further increasing cytokine production, enhancing pancreatic leukocyte sequestration and accelerating acinar cell apoptosis, ultimately leading to a systemic inflammatory response [8,9]. It has been demonstrated that inhibition of NF-κB activation reduces acinar cell damage and decreases the severity of pancreatitis [10]. Recently, anti-TNF-α treatment in experimental pancreatitis was reported to be of benefit, especially when administered early [11]. However, its effect on established necrotizing pancreatitis is not known.

The protein C pathway serves as a major system for controlling thrombosis, limiting inflammatory responses, and potentially decreasing endothelial cell apoptosis in response to inflammatory cytokines [12]. Recombinant human activated protein C (APC) is known to inhibit coagulation and inflammation, and to promote fibrinolysis in patients with severe sepsis [13]. Binding of APC to the endothelial cell protein C receptor results in a number of actions, including increased activity of APC itself and inhibition of both NF-κB and apoptosis [14].

Edema progresses to necrosis in about 20% of patients with acute pancreatitis [15]. The pancreas is infected in 40–70% of patients with necrotizing pancreatitis, and the mortality rate may be up to 40% when the necrotic tissue becomes superinfected [16]. The most important cause of death in necrotizing pancreatitis is secondary infections, which generally result from translocation of enteric bacteria from the intestine via mainly lymphatic, hematogenous, or transmural routes [17]. On the other hand, prophylactic antibiotic therapy was not found to decrease mortality in controlled clinical trials [18]. Although selective gut decontamination and, to some extent, enteral nutrition were shown to decrease infectious complications [19,20], no specific agent that can strengthen the gut barrier or inhibit translocation of micro-organisms from the gut lumen has yet been identified.

Our aim in the present study was to investigate the effects of recombinant human APC on the progression of experimental ANP. Considering its significant role in inflammatory responses, we hypothesized that APC may alter the degree of local inflammation, development of necrosis and bacterial contamination, and thus the severity of acute pancreatitis.

## Materials and methods

The experiment was approved by the Institutional Animal Use and Care Committee of the Gülhane Medical Academy and was performed in accordance with the US National Institutes of Health guidelines for the care and handling of animals.

### Animals

Male Sprague–Dawley rats weighing 280–350 g were obtained from the Gülhane School of Medicine Research Center (Ankara, Turkey). Before the experiment the animals were fed standard rat chow, were given free access to water, and were housed in metabolic cages with controlled temperature and 12-hour light–dark cycles for at least 1 week.

### Induction of pancreatitis

Anesthesia was induced in rats via inhalation of 250 ml sevoflurane liquid (Abbott, Istanbul, Turkey). Laparotomy was performed through a midline incision. After cannulation of the common biliopancreatic duct with a 28-gauge, 0.5 inch microfine catheter, a microaneurysm clip was placed on the bile duct below the liver and another around the common biliopancreatic duct at its entry into the duodenum to avoid reflux of enteric contents into the duct. Then, 1 ml/kg of 5% sodium taurocholate (Sigma, St. Louis, MO, USA) was slowly infused into the common biliopancreatic duct. The infusion pressure was kept below 30 mmHg, as measured using a mercury manometer. When the infusion was complete, the two microclips were removed and the abdomen was closed in two layers. All procedures were performed using sterile technique.

### Study protocol

After the stabilization period, 45 male rats were randomly divided into three groups. Rats in group I (control group; *n *= 15) underwent laparotomy with manipulation of the pancreas (sham procedure) and received 10 ml/kg saline intravenously (single dose). Groups II and III underwent laparotomy with induction of ANP. Rats in group II (positive control; *n *= 15) received saline, as in group I but 6 hours after induction of ANP. Rats in group III (treatment group; *n *= 15) received 100 mg/kg recombinant human APC (Drotrecogin alfa [activated]; Xigris; Lilly, Istanbul, Turkey) intravenously (single dose) 6 hours after induction of ANP. Twenty-four hours after induction of ANP, all surviving animals were killed by intracardiac infection of pentobarbital (200 mg/kg). Blood samples were taken from the heart before the animals were killed in order to measure serum amylase, TNF-α, and IL-6. Animals that died before the end of the study (four in group II and two in group III) were excluded from the analysis.

### Histopathologic analysis

A portion of the pancreas from the same anatomical location in each rat, including the main pancreatic duct, was fixed in 10% neutral buffered formalin and embedded in paraffin. One paraffin section stained with hematoxylin and eosin was examined for each pancreas. Two pathologists, who were blinded to the treatment protocol, scored the tissues with respect to edema, acinar necrosis, inflammatory infiltrate, hemorrhage, fat necrosis, and perivascular inflammation in 20 fields. The scores for each histological examination were summed, yielding a maximum score of 24, as defined by Schmidt and coworkers [21].

### Amylase measurement

A Hitachi 917 autoanalyzer (Boehringer Mannheim, Mannheim, Germany) was used in the amylase assay.

### Tumor necrosis factor-α and interleukin-6 assays

Blood was collected and centrifuged (3000 rpm for 5 min). The serum was stored at -40°C. TNF-α and IL-6 were measured in serum samples using quantitative sandwich enzyme-linked immunosorbent assay kits (R&D Systems Inc., Minneapolis, MN, USA).

### Quantitative cultures and bacterial identification

Tissue specimens taken from mesenteric lymph nodes (MLNs) and one portion of the pancreas with macroscopic necrosis were harvested for culture. Each sample was weighed and homogenized. Afterward, the homogenates were diluted serially, quantitatively plated in duplicate on phenylethyl alcohol and MacConkey II agar, and then incubated aerobically at 37°C for 24 hours. Bacterial counts were expressed as colony-forming units/g tissue, and counts of 1000 colony-forming units/g and higher were considered to represent a positive culture. Gram-negative bacteria were identified using the API-20E system (BioMerieux Vitek, Hazelwood, MO, USA). Gram-positive bacteria were identified to the genus level using standard microbiologic methods.

### Statistical analysis

Results are expressed as mean ± standard error of the mean. Translocation incidence was evaluated by Fisher's exact test. The significance of differences in total histopathologic scores, serum amylase activities, and cytokine levels were assessed using one-way analysis of variance and Tukey HSD as *post hoc *tests. Detailed histopathologic scores (e.g. edema and acinar necrosis) were assessed using the Kruskal–Wallis test, and subgroup analyses were conducted using the Mann–Whitney U-test. *P *< 0.05 was considered statistically significant. All statistical measurements were done using SPSS PC version 9.05 (SPSS Inc., Chicago, IL, USA).

## Results

**Figure 1:**
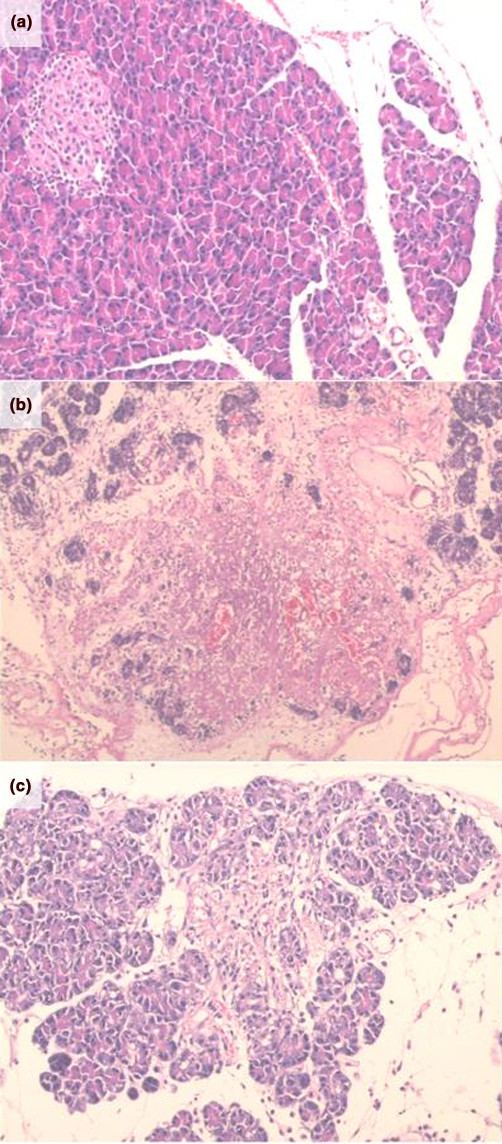
Histology samples from the three groups. **(a) **Normal pancreatic histology in group I (the sham operated group). **(b) **Light micrograph showing severe and extensive parenchymal necrosis, with few normal acinar cells in group II (the positive control group). **(c) **Light micrograph showing mild edema, parenchymal focal necrosis, and inflammation in group III (the activated protein C treated group). All samples were stained with hematoxylin and eosin, and the original magnification for each image is 50×.

Rats with ANP had extensive parenchyma and fat necrosis, and polymorphonuclear leukocyte infiltration on histologic examination. The total histopathologic score was significantly reduced in group III (10.31 ± 0.47) compared with group II (14.00 ± 0.52; *P *< 0.001). Although there were marked improvements in pancreatic tissue edema, inflammatory infiltration, fat necrosis, acinar necrosis scores, and perivascular inflammation in APC-treated group III compared with saline-treated group II, there was no significant difference in hemorrhage scores between the two groups (Fig. 1). Histopathologic findings in the groups are summarized in Table 1.

### Serum amylase and cytokines assay

Serum amylase, TNF-α, and IL-6 levels in group I (the sham group) were significantly lower than in the other two groups. Significant reductions were found in serum levels of amylase (*P *< 0.001), TNF-α (P < 0.04), and IL-6 (*P *< 0.001) in group III (the APC-treated group) compared with group II (the positive control group; Table 2).

### Bacterial translocation

**Figure 2:**
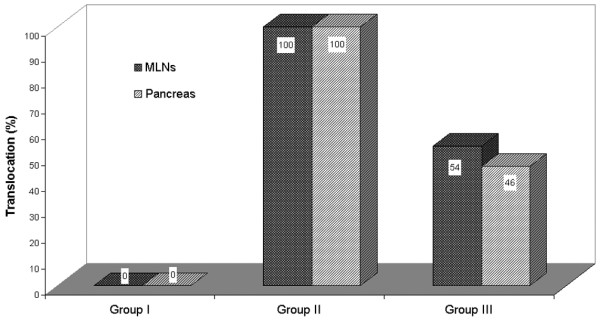
Incidences of bacterial translocation to mesenteric lymph nodes (MLNs) and pancreas. Group I, sham operated group; group II, positive control group; group III, activated protein C treated group.

Bacteria were cultured from MLNs and pancreatic necrotic tissues in all 11 animals in saline-treated group II. In APC-treated group III, bacterial cultures from MLN samples and pancreatic necrotic tissue samples were positive in seven (54%) and six (46%) of the 13 animals, respectively. MLN and pancreatic tissue infection rates in group III were significantly lower than in group II (*P *< 0.02 and *P *< 0.007, respectively). The incidences of bacterial translocation in the three groups are summarized in Fig. 2. *Escherichia coli *was the most commonly isolated bacteria. Other bacteria isolated from MLNs and pancreatic tissues are listed in Table 3. No organisms were found in either MLNs or pancreatic tissues in rats from group I (sham operated).

## Discussion

Acute pancreatitis represents a severe form of inflammation that often leads to severe damage to the gland. Progression from edematous to necrotizing pancreatitis – a process that usually determines the patients' prognosis – is mediated by NF-κB [7]. In the present study, plasma IL-6 and TNF-α levels, together with amylase, were significantly increased after induction of ANP. Stimulation of production of either acute phase proteins and adhesion molecules or several inflammatory cytokines, including TNF-α, IL-1β and IL-6, occurs after NF-κB activation in acute pancreatitis [7]. However, we observed that amylase, and plasma IL-6 and TNF-α levels were all significantly decreased in APC-treated animals.

APC has been shown to inhibit production of TNF-α by decreasing activation of NF-κB [22]. In contrast to many immunomodulatory agents previously tested clinically, recombinant human APC was found to be significantly beneficial in the PROWESS (Recombinant Human Activated Protein C Worldwide Evaluation in Severe Sepsis) study and was approved by the US Food and Drug Administration for use in patients with severe sepsis and septic shock [23,24]. A significant decrease in protein C concentrations was found during the initial phase of experimental ANP [25]. Furthermore, drotrecogin alfa (activated) treatment was recently reported to improve progression of severe sepsis after ANP in two cases [26]. Based on these data and those presented above, APC replacement may interrupt, at least partly, the pathophysiological cascade of inflammation and related events during ANP.

We found significant improvements in pancreatic histology after treatment with recombinant APC. Edema, acinar cell necrosis, fat necrosis, and perivascular inflammation, which occur in almost all inflammatory processes in any organ, resolved in pancreatic tissues from animals treated with APC. However, although we know that the decrease in APC occurs during the initial period of pancreatitis, we only studied its effects in established ANP because we believe that an experimental model should simulate the situation in humans. Indeed, clinically, only a small number of patients with acute pancreatitis present during the early stages of disease. Therefore, the results of the present study are relevant to clinical necrotizing pancreatitis in humans. The concept of administering APC to patients with the disease immediately after the diagnosis is established is rational and should be the focus of research. Nevertheless, more experimental and clinical evidence is needed if we are to evaluate the value of such prophylactic use of APC.

Little is known about effects on the coagulation system in ANP. Because the degree of hemorrhage affects the extent of local and systemic complications in ANP, maintenance of a normal coagulation system in the pancreatic microcirculation in order to prevent thrombosis or bleeding is a desirable objective. Protein C is a critical participant in normal coagulation mechanisms. One interesting finding in the present study was the similarity in hemorrhage scores between groups II and III. In comparison with control animals, APC neither decreased nor increased the incidence of hemorrhagic fields in tissue samples. This not only may refect the anticoagulant effect of APC but also suggests that the coagulation system in pancreas remains intact, even with the organ in a necrotic state.

Evaluation of bacterial translocation after APC treatment was another aim of the study. We found lesser MLN and pancreatic bacterial contamination in APC-administered rats than in control animals. The impact of superinfection of the pancreas is summarized above. The decreased contamination rates may reflect APC-related improvements in gut mucosa. Contamination of necrotic tissues occurs primarily because of translocation of enteric micro-organisms [27]. Our study does not indicate any direct effect of APC on intestinal mucosa, although many factors have been reported to underlie bacterial translocation [27], including intestinal mucosal injury, cecal bacterial overgrowth, decreased gut motility, and compromised host immune functions. Failure of the gut to act as a barrier against bacterial translocation as a result of nitric oxide (NO)-dependent mechanisms [28] has been accepted as one of the most potent origins of sepsis and subsequent organ failure after pancreatitis [29], and inhibition of inducible NO synthase was shown to decrease the incidence of bacterial translocation [30]. Isobe and coworkers [31] found an inhibitory effect of APC on inducible NO synthase induction by decreasing TNF-α production in rats with endotoxin-induced hypotension; a similar action of APC in the impaired intestinal mucosa of the rats with ANP might have been at work in the present study. Future studies addressing the association between APC and NO-dependent damage in the intestine following ANP induction will help to identify a possible second role of APC in the pathogenesis of the disease.

## Conclusion

APC improved pancreatic histology and decreased the incidence of bacterial translocation from the intestine in rats with experimental ANP. APC and its reduction appear to play an important role in the pathogenesis of this life-threatening disease. Therefore, the effects of replacement of this mediator in ANP should be a focus of future investigations.

## Key messages

• 	Decrease in APC is important in the pathogenesis of acute pancreatitis related systemic complications.

• 	Replacement with recombinant APC improved local injury and markers of systemic inflammation, and decreased bacterial translocation from the gut in experimental ANP.

• 	APC administration may be an alternative treatment option in patients with ANP.

## Abbreviations

ANP = acute necrotizing pancreatitis; APC = activated protein C; IL = interleukin; MLN = mesenteric lymph node; NF-κB = nuclear factor-κB; NO = nitric oxide; TNF = tumor necrosis factor.

## Competing interests

The author(s) declare that they have no competing interests.

## Authors' contributions

LY and MRM conceived the study. BC performed statistical analysis. ATI and SA performed the surgical procedures. NM and SD carried out histological analysis. MO and IT carried out microbiological, amylase and cytokine assays. TU was involved in drafting and revising the manuscript. All authors read and approved the final manuscript.

## Figures and Tables

**Table 1:** Histopathologic scores in the three groups

Feature	Group I (sham)	Group II (control)	Group III (treatment)	*P *(group II versus group III)
Edema	0.67 ± 0.13	3.09 ± 0.16	2.38 ± 0.14	<0.007^a^
Acinar necrosis	0.07 ± 0.06	1.82 ± 0.12	1.08 ± 0.08	<0.001^a^
Inflammatory infiltrate	0.73 ± 0.12	2.91 ± 0.16	2.00 ± 0.16	<0.002^a^
Hemorrhage	0.27 ± 0.12	2.45 ± 0.16	2.08 ± 0.18	NS^a^
Fat necrosis	0.13 ± 0.09	1.82 ± 0.12	1.31 ± 0.13	<0.02^a^
Perivascular Inflammation	0.13 ± 0.09	1.91 ± 0.09	1.38 ± 0.14	<0.02^a^
Histopathologic score	2.00 ± 0.28	14.00 ± 0.52	10.31 ± 0.47	<0.001^b^

Values are expressed as mean score ± standard error of the mean. ^a^By Mann–Whitney U test.

^b^By Tukey HSD. NS, not significant.

**Table 2:** Serum amylase, tumor necrosis factor-α, and interleukin-6 levels of groups

	Group I (sham)	Group II (control)	Group III (treatment)	*P *(group II versus group III)
Amylase (pg/ml)	597.2 ± 22.0	1848.3 ± 96.2	1236.1 ± 69.9	<0.001^a^
TNF-α (pg/ml)	63.4 ± 5.1	114.4 ± 7.9	88.5 ± 7.7	<0.04^a^
IL-6 (pg/ml)	201.9 ± 17.2	1391.8 ± 106.6	816.2 ± 73.1	<0.001^a^

Values are expressed as mean ± standard error of the mean. ^a^By Tukey HSD. IL, interleukin; TNF, tumor necrosis factor.

**Table 3:** Bacteria isolated from mesenteric lymph node and pancreatic tissue samples

Bacteria	Group II (*n *= 11)		Group III (*n *= 13)	
	
	MLNs	Pancreas	MLNs	Pancreas
*Escherichia coli*	7	8	6	5
*Enterococcus *sp.	3	2	1	1
*Staphylococcus *sp.	1	1	0	0
*Klebsiella oxytoca*	2	1	1	0
*Proteus*	1	0	0	0
Polimicrobial	2	1	1	0

MLN, mesenteric lymph node.
